# NLRP3 Deficiency Reduces Macrophage Interleukin-10 Production and Enhances the Susceptibility to Doxorubicin-induced Cardiotoxicity

**DOI:** 10.1038/srep26489

**Published:** 2016-05-26

**Authors:** Motoi Kobayashi, Fumitake Usui, Tadayoshi Karasawa, Akira Kawashima, Hiroaki Kimura, Yoshiko Mizushina, Koumei Shirasuna, Hiroaki Mizukami, Tadashi Kasahara, Naoyuki Hasebe, Masafumi Takahashi

**Affiliations:** 1 Division of Inflammation Research, Center for Molecular Medicine Jichi Medical University, 3311-1, Yakushiji, Shimotsuke, Tochigi, Japan; 2 Division of Genetic Therapeutics, Jichi Medical University, 3311-1, Yakushiji, Shimotsuke, Tochigi, Japan; 3 Department of Medicine, Division of Cardiovascular, Respiratory and Neurology, Asahikawa Medical University, 2-1-1-1 Midorigaoka Higashi, Asahikawa, Hokkaido, Japan

## Abstract

NLRP3 inflammasomes recognize non-microbial danger signals and induce release of proinflammatory cytokine interleukin (IL)-1β, leading to sterile inflammation in cardiovascular disease. Because sterile inflammation is involved in doxorubicin (Dox)-induced cardiotoxicity, we investigated the role of NLRP3 inflammasomes in Dox-induced cardiotoxicity. Cardiac dysfunction and injury were induced by low-dose Dox (15 mg/kg) administration in NLRP3-deficient (NLRP3^−/−^) mice but not in wild-type (WT) and IL-1β^−/−^ mice, indicating that NLRP3 deficiency enhanced the susceptibility to Dox-induced cardiotoxicity independent of IL-1β. Although the hearts of WT and NLRP3^−/−^ mice showed no significant difference in inflammatory cell infiltration, macrophages were the predominant inflammatory cells in the hearts, and cardiac IL-10 production was decreased in Dox-treated NLRP3^−/−^ mice. Bone marrow transplantation experiments showed that bone marrow-derived cells contributed to the exacerbation of Dox-induced cardiotoxicity in NLRP3^−/−^ mice. *In vitro* experiments revealed that NLRP3 deficiency decreased IL-10 production in macrophages. Furthermore, adeno-associated virus-mediated IL-10 overexpression restored the exacerbation of cardiotoxicity in the NLRP3^−/−^ mice. These results demonstrated that NLRP3 regulates macrophage IL-10 production and contributes to the pathophysiology of Dox-induced cardiotoxicity, which is independent of IL-1β. Our findings identify a novel role of NLRP3 and provided new insights into the mechanisms underlying Dox-induced cardiotoxicity.

Doxorubicin (Dox; Adriamycin) is one of the most widely used and successful drugs in cancer chemotherapy. Although it is highly effective in a wide range of cancers, including leukemia, carcinoma, and soft tissue sarcomas, its clinical use is limited because of its adverse effects, especially myocardial injury and subsequent heart failure^1^. The mechanisms of Dox-induced cardiotoxicity include oxidative stress, induction of apoptosis, and intracellular calcium dysregulation; however, the proposed mechanisms remain controversial^2^,^3^. Recently, the beneficial effects of deletion of Toll-like receptors (TLRs), which are the key components of innate immune responses, have been demonstrated. For instance, Riad *et al.*^4^ reported that TLR-4 deficient (TLR4^−/−^) mice show improved cardiac dysfunction with reduced oxidative stress and inflammatory responses after Dox administration. In addition, Nozaki *et al.*^5^ observed that cardiac dysfunction and apoptotic cell death were attenuated in Dox-treated TLR2^−/−^ mice. Since microbial infection is not involved in the pathogenesis of Dox-induced cardiotoxicity, these findings suggest the importance of sterile inflammation in the process of Dox-induced cardiotoxicity. However, little is known about the role and mechanisms of sterile inflammation in the pathophysiology of Dox-induced cardiotoxicity.

Increasing evidence indicates that some types of sterile inflammation are mediated through inflammasomes. The inflammasomes are intracellular multiprotein complexes and regulate the secretion of proinflammatory cytokine interleukin (IL)-1β^6^,^7^,^8^. Although several types of inflammasomes have been identified to date, the nucleotide-binding oligomerization domain-like receptor (NLR) family pyrin domain containing 3 (NLRP3, also known as NALP3 and cryopyrin) inflammasomes have been implicated in sterile inflammation. NLRP3 inflammasomes are composed of NLRP3, apoptosis-associated speck-like protein containing a caspase recruitment domain (ASC), and caspase-1 and induce caspase-1 activation. Because caspase-1 was previously known as an IL-1β-converting enzyme^9^, its activation processes pro-IL-1β to its mature form and induces IL-1β secretion, leading to inflammatory responses and tissue damage. We recently demonstrated the importance of NLRP3 inflammasomes in the pathogenesis of certain cardiovascular diseases such as myocardial ischemia-reperfusion (I/R), atherosclerosis, vascular injury, and abdominal aortic aneurysm^10^,^11^,^12^,^13^. With respect to the relationship between NLRP3 inflammasomes and Dox, Sauter *et al.*^14^ reported that Dox activated NLRP3 inflammasomes and induced processing of pro-IL-1β in murine bone marrow-derived macrophages *in vitro*. In addition, Zhu *et al.*^15^ reported that treatment with recombinant human IL-1 receptor antagonist prevented Dox-induced cardiotoxicity. However, the role of NLRP3 inflammasomes in Dox-induced cardiotoxicity is unknown. Therefore, we hypothesized that NLRP3 inflammasomes mediate Dox-induced cardiotoxicity. To test this hypothesis, we used NLRP3^−/−^ and IL-1β^−/−^mice and unexpectedly found that NLRP3 deficiency exacerbated Dox-induced cardiac dysfunction and injury independently of IL-1β. Furthermore, we identified that the exacerbation of cardiotoxicity by NLRP3 deficiency was mediated by decreased IL-10 production in macrophages. These findings demonstrate a novel role of NLRP3 in the pathophysiology of Dox-induced cardiotoxicity and suggest that NLRP3 and IL-10 are therapeutic targets for the treatment of Dox-induced cardiotoxicity.

## Results

### NLRP3 deficiency exacerbates Dox-induced cardiotoxicity

**Figure 1:**
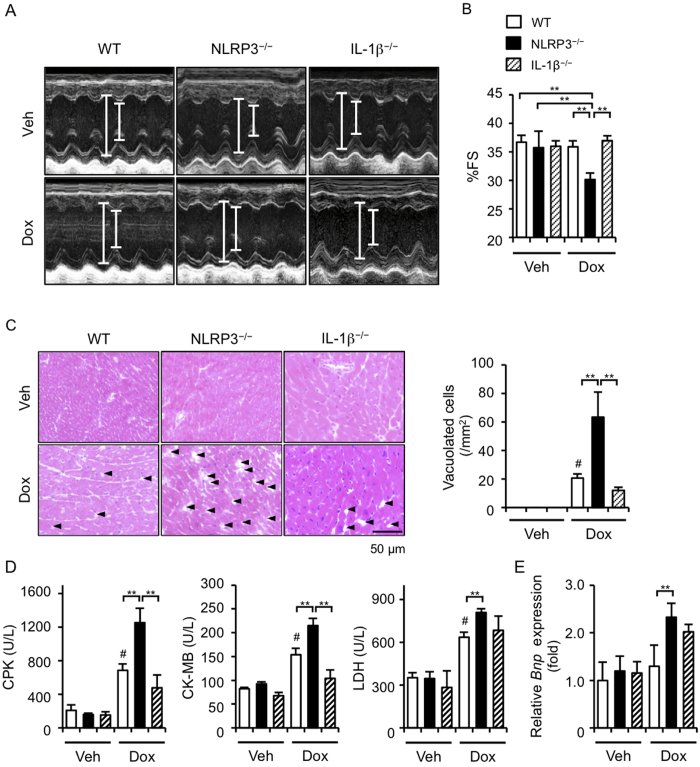
NLRP3 deficiency exacerbates Dox-induced cardiac injury. (**A**,**B**) Echocardiography was performed in WT, NLRP3^−/−^, and IL-1β^−/−^mice 5 days after Dox (15 mg/kg) or vehicle (Veh) treatment. Two-dimensional M-mode echocardiograms are shown (**A**). Cardiac function (%FS) was assessed (**B**) (n = 6–10). (**C–E**) Mice were sacrificed 5 days after Dox or Veh treatment. (**C**) The heart sections were obtained and stained with HE. The vacuolated cardiomyocytes were quantified (n = 4 for each). (**D**) Plasma levels of CPK, CK-MB, and LDH were assessed (n = 5 for each). (**E**) Heart *Bnp* mRNA levels were assessed by real-time RT-PCR analysis (n = 4 for each). Data are expressed as the mean ± SEM. **p < 0.01. ^#^*p* < 0.05 vs. Veh treatment (WT).

To investigate the effect of Dox on cardiotoxicity, we first administered 20 mg/kg Dox in WT and NLRP3^−/−^ mice because this dose of Dox has been shown to induce cardiac dysfunction and injury in mice^4^,^5^. Cardiac dysfunction (decreased %FS) and injury (increased levels of plasma CPK, CK-MB, and LDH) were observed in Dox-treated WT and NLRP3^−/−^ mice at similar extents (Supplementary Fig. S1). However, histological analysis revealed that the number of vacuolated cardiomyocytes tended to be increased in the hearts of NLRP3^−/−^ mice, compared to WT mice. This result prompted us to examine the effects of lower dose Dox. We unexpectedly found that treatment with 15 mg/kg Dox induced cardiac dysfunction in NLRP3^−/−^ mice, but not in WT and IL-1β^−/−^ mice (Fig. 1A,B). The number of vacuolated cardiomyocytes and plasma levels of CPK, CK-MB, and LDH were significantly increased in WT mice and were further strikingly increased in NLRP3^−/−^ mice, compared to WT and IL-1β^−/−^ mice (Fig. 1C,D). In addition, a similar trend of *Bnp* mRNA expression, a marker of cardiomyocytes injury, in the hearts among these mice was observed (Fig. 1E). These findings suggest that NLRP3 deficiency enhances the susceptibility to Dox-induced cardiotoxicity independently of IL-1β.

### NLRP3 deficiency has no effect on inflammatory cell infiltration but decreases IL-10 production

To explore the mechanism by which NLRP3 deficiency enhances the susceptibility to Dox-induced cardiotoxicity, we next assessed apoptosis, which has been shown to be involved in Dox-induced cardiotoxicity^2^,^5^. TUNEL staining showed that apoptotic cell death was rarely detected at this dose of Dox, and there was no significant difference in cell death between WT and NLRP3^−/−^ mice (Supplementary Fig. S2A). Consistent with this, the ratio of *Bax/Bcl-2*, a key factor in the regulation of apoptosis, did not differ in the hearts between WT and NLRP3^−/−^ mice (Supplementary Fig. S2B).

**Figure 2:**
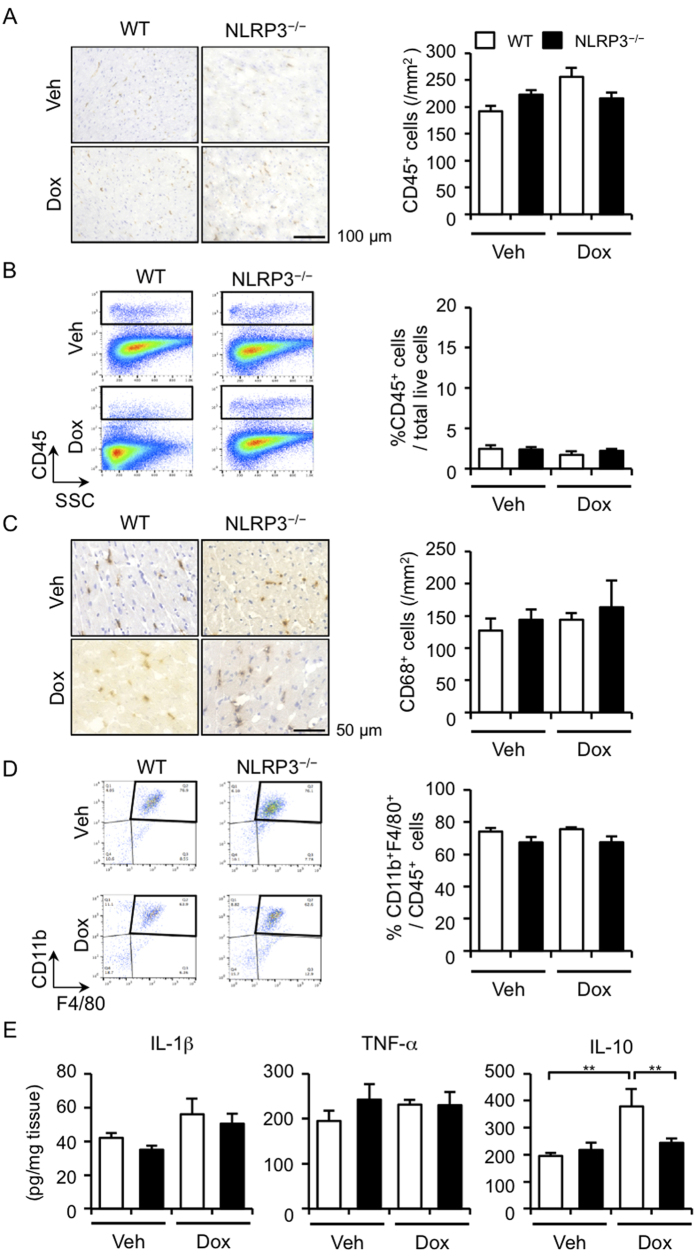
NLRP3 deficiency has no effect on inflammatory cell infiltration but decreases IL-10 production. The heart samples were obtained from WT and NLRP3^−/−^ mice 5 days after Dox or vehicle treatment. (**A**) The heart sections were stained immunohistochemically for CD45. The number of CD45^+^ cells was quantified (n = 5 for each). (**B**) The number of leukocytes (CD45^+^ cells) in the heart was analyzed by flow cytometry (n = 5 for each). (**C**) The heart sections were stained immunohistochemically for CD68. The number of macrophages (CD68^+^ cells) was quantified (n = 5 for each). (**D**) The number of macrophages (CD11b^+^F4/80^+^ cells) in the heart was analyzed by flow cytometry (n = 5 for each). (**E**) The protein levels of IL-1β, TNF-α, and IL-10 in the heart were assessed (n = 5 for each). Data are expressed as the mean ± SEM. **p < 0.01.

Because we have recently demonstrated that NLRP3 regulated the migration of inflammatory cells and contributes to the pathogenesis of hepatic I/R injury and hyperoxia-induced lung injury independently of IL-1β^16^,^17^. We assessed inflammatory cell infiltration in the hearts. Immunohistochemical analysis for the pan-leukocyte marker, CD45, and the macrophage marker, CD68, showed that Dox treatment did not induce inflammatory cell infiltration in the hearts of the WT and NLRP3^−/−^ mice, and there was no difference in the infiltration between these mice (Fig. 2A,C). We further confirmed the profile of CD45^+^ cells and CD11b^+^F4/80^+^ cells (macrophages) infiltrated in the hearts using flow cytometry analysis (Fig. 2B,D). We analyzed the composition of inflammatory cells in the hearts and found that compared with neutrophils (Ly6G^+^ CD45R^−^), T cells (CD4^+^ and CD8^+^), and B cells (CD19^+^), macrophages (CD11b^+^F4/80^+^) were present predominantly in the hearts (Supplementary Fig. S3). We also assessed the production of inflammatory cytokines in the heart tissues and showed that Dox treatment failed to increase the pro-inflammatory cytokines IL-1β and TNF-α in WT and NLRP3^−/−^ mice (Fig. 2E). Interestingly, however, a pivotal anti-inflammatory cytokine IL-10 was significantly increased in the hearts of Dox-treated WT mice, but no increase of IL-10 was detected in the hearts of Dox-treated NLRP3^−/−^ mice. These results suggest that the anti-inflammatory cytokine IL-10 is involved in the exacerbation of cardiotoxicity in NLRP3^−/−^ mice.

### NLRP3 in bone marrow cells contributes to Dox-induced myocardial injury

**Figure 3:**
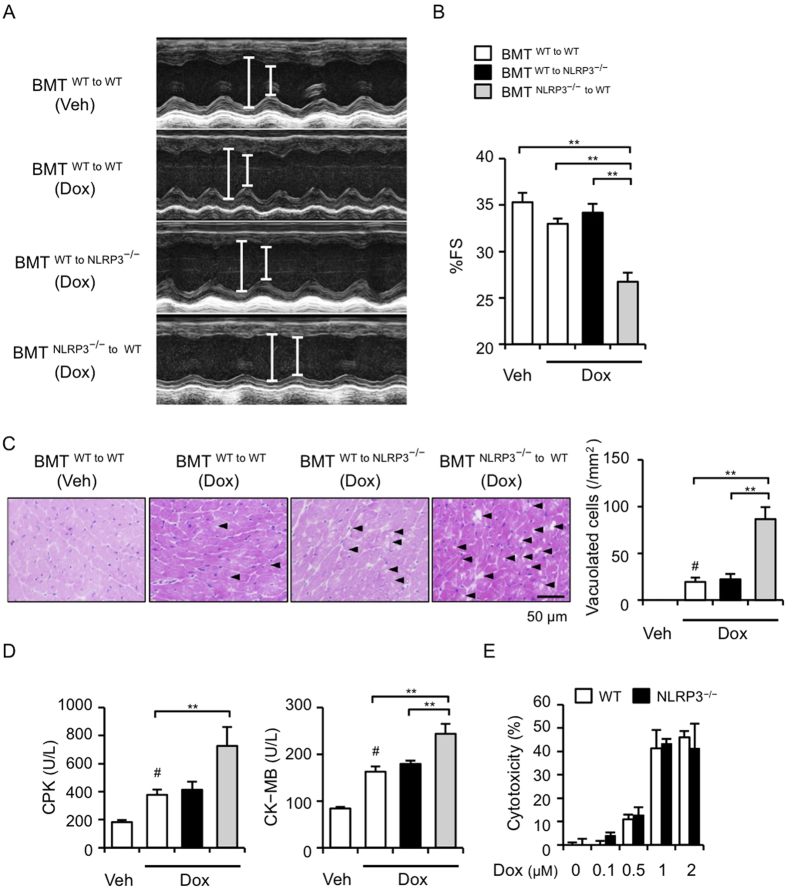
NLRP3 in bone marrow cells contributes to Dox-induced myocardial injury. BMT^WT to WT^, BMT ^WT to NLRP3−/−^, and BMT^NLRP3−/− to WT^ mice were developed, and treated with Dox or Veh 8 weeks after BMT. Echocardiography was performed 5 days after Dox or Veh treatment. Two-dimensional M-mode echocardiograms are shown (**A**). Cardiac function (%FS) was assessed (**B**) (n = 6–10). (**C,D**) Mice were sacrificed 5 days after Dox or Veh treatment. (**C**) The heart sections were obtained and stained with HE. The vacuolated cardiomyocytes were quantified (n = 4 for each). (**D**) Plasma levels of CPK and CK-MB were assessed (n = 5 for each). (**E**) Primary cardiomyocytes were isolated from WT and NLRP3^−/−^ mice, and treated with Dox (0.1–2.0 μM) for 24 h. Cytotoxicity was assessed by LDH activity (n = 6 for each). Data are expressed as the mean ± SEM. **p < 0.01, and ^#^*p* < 0.05 vs. Veh treatment (BMT^WT to WT^).

Since IL-10 is produced predominantly by inflammatory cells^18^, we produced three types of bone marrow transplantation (BMT) mice (BMT^WT to WT^, and BMT^WT to NLRP3−/−^, and BMT^NLRP3*−/−* to WT^ mice) and investigated the contribution of bone marrow-derived inflammatory cells to Dox-induced cardiotoxicity. Cardiac dysfunction and injury became apparent in BMT^NLRP3−/− to WT^ mice, compared with those in BMT^WT to WT^ and BMT^WT to NLRP3−/−^ mice (Fig. 3A–D). In addition, IL-10 levels in the hearts of Dox-treated BMT^NLRP3*−/−* to WT^ mice were lower than those of Dox-treated BMT^WT to WT^ and BMT^WT to NLRP3−/−^ mice (Supplementary Fig. S4). No significant levels of IL-10 were detected in the plasma of these BMT mice (data not shown). Supporting these results, *in vitro* experiments showed no difference in Dox-induced cardiotoxicity in primary cardiomyocytes between WT and NLRP3^−/−^ mice (Fig. 3E). These results indicate that bone marrow-derived inflammatory cells contribute to the exacerbation of Dox-induced cardiotoxicity in NLRP3^−/−^ mice.

### NLRP3 deficiency exhibites decreased IL-10 production in macrophages

**Figure 4:**
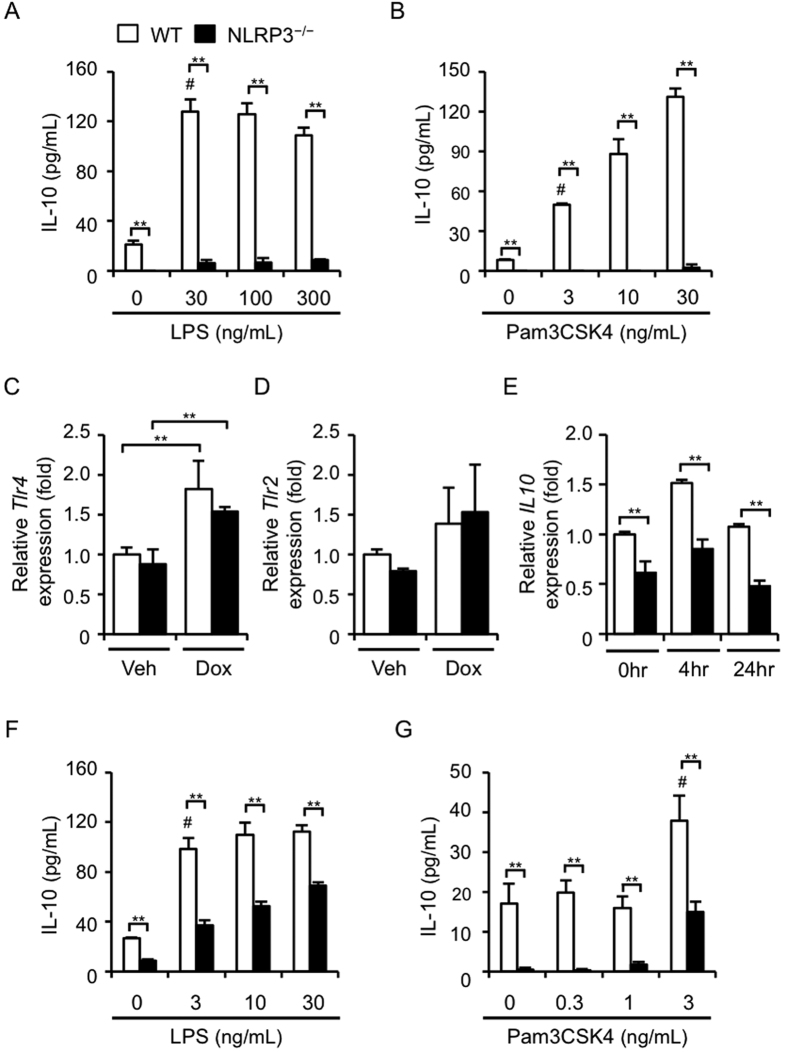
NLRP3 deficiency causes decreased IL-10 production in macrophages. (**A**,**B**) Primary macrophages derived from WT and NLRP3^−/−^ mice were stimulated with LPS (30–300 ng/mL) or Pam3CSK4 (3–30 ng/mL) for 24 h at the concentrations indicated. The IL-10 protein levels in the supernatants were assessed. (**C,D**) Primary WT and NLRP3^−/−^ macrophages were treated with Dox (2 μM) or Veh for 24 h. The mRNA levels of *Tlr4* and *Tlr2* were assessed (n = 5 for each). (**E**) Primary WT and NLRP3^−/−^ macrophages were treated with Dox or Veh for the indicated periods. The *Il10* mRNA levels were assessed (n = 5 for each). (**F,G**) Splenocytes isolated from WT and NLRP3^−/−^ mice were stimulated with LPS (3–10 ng/mL) or Pam3CSK4 (0.3–3 ng/mL) for 24 h at the concentrations indicated. Data are expressed as the mean ± SEM. **p < 0.01. ^#^*p* < 0.05 vs. no LPS treatment (WT).

Our results indicate that macrophages are the predominant inflammatory cells in the hearts and that IL-10 is involved in the exacerbation of Dox-induced cardiotoxicity. In addition, previous studies have suggested that TLR2/4 signaling is involved in Dox-induced cardiotoxicity^4^,^5^. These findings prompted us to examine whether NLRP3 deficiency could influence TLR-mediated IL-10 production in primary macrophages. Consistent with the results of a previous study^18^, LPS (TLR4 ligand; 30–300 ng/mL) clearly stimulated IL-10 production in WT macrophages (Fig. 4A). Notably, LPS-induced IL-10 production was markedly inhibited in NLRP3^−/−^ macrophages. Similar results were obtained when the macrophages were stimulated with Pam3CSK4 (TLR1/2 ligand; 3–30 ng/mL; Fig. 4B). To exclude the possibility that the expression of TLR2/4 could be downregulated in NLRP3^−/−^ macrophages, we determined the levels of *Tlr4* and *Tlr2* mRNA and ascertained no significant differences in *Tlr4* and *Tlr2* levels between WT and NLRP3^−/−^ macrophages (Fig. 4C,D). Furthermore, *Il10* mRNA expression was also inhibited in NLRP3^−/−^ macrophages, compared with the expression in WT macrophages (Fig. 4E). Similar results were obtained using primary splenocytes isolated from WT and NLRP3^−/−^ mice (Fig. 4F,G).

Following TLR stimulation, ERK1/2, p38, and NF-κB have been shown to be common upstream pathways for IL-10 induction in macrophages^18^. To investigate the mechanisms of decreased IL-10 production in NLRP3^−/−^ macrophages, we assessed the activation of these upstream pathways. Western blot analysis showed marked phosphorylation of ERK1/2 and p38 as well as degradation of IκBα in response to LPS in WT macrophages (Supplementary Fig. S5A). Similar patterns of their phosphorylation and degradation were observed in NLRP3^−/−^ macrophages. Furthermore, immunofluorescence staining showed that LPS-induced nuclear translocation of p65 did not differ between WT and NLRP3^−/−^ macrophages (Supplementary Fig. S5B). These findings suggest that NLRP3 regulates IL-10 production independently of ERK1/2, p38, and NF-κB.

### AAV-mediated IL-10 overexpression restores the exacerbation of Dox-induced cardiotoxicity

**Figure 5:**
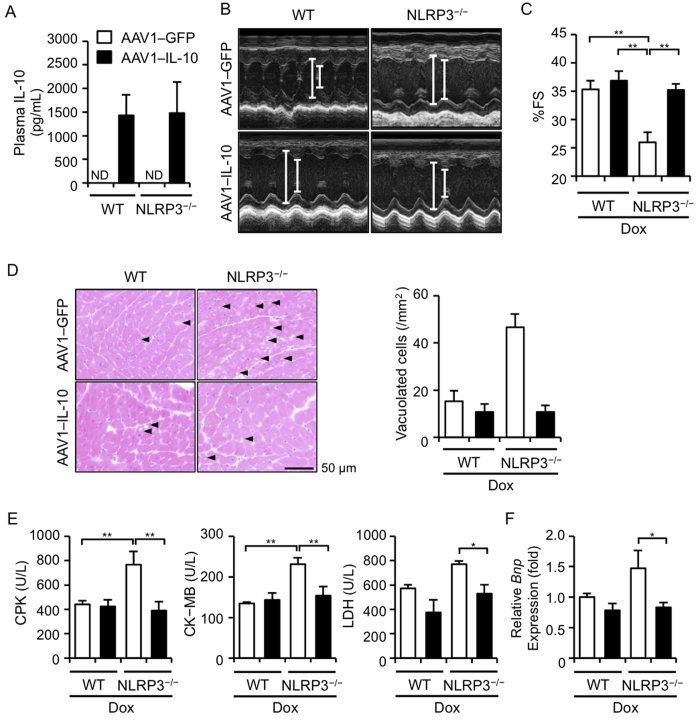
AAV-mediated IL-10 overexpression restores Dox-induced cardiotoxicity. WT and NLRP3^−/−^ mice were injected intramuscularly with AAV1-GFP and AAV1-IL-10. (**A**) The IL-10 protein levels were assessed at 14 days after the injection (n = 8–9 for each). ND indicates not detected. (**B–F**) WT and NLRP3^−/−^ mice were treated with Dox or Veh 14 days after the AAV injection. (**B,C**) Echocardiography was performed 5 days after Dox or Veh treatment. Two-dimensional M-mode echocardiograms are shown (**B**). Cardiac function (%FS) was assessed (**C**) (n = 6–10). (**D–F**) Mice were sacrificed 5 days after Dox or Veh treatment. (**D**) The heart sections were obtained and stained with HE. The vacuolated cardiomyocytes were quantified (n = 4 for each). (**E**) Plasma levels of CPK, CK-MB, and LDH were assessed (n = 5 for each). (**F**) Heart *Bnp* mRNA levels were assessed by real-time RT-PCR analysis (n = 4 for each). Data are expressed as the mean ± SEM. *p < 0.05 and **p < 0.01.

Our results strongly suggest that IL-10 is a key mediator for ameliorating the exacerbation of Dox-induced cardiotoxicity in NLRP3^−/−^ mice. Therefore, we finally determined whether IL-10 overexpression could restore this cardiac phenotype in NLRP3^−/−^ mice. To induce IL-10 overexpression, we used AAV vectors because they are efficient vehicles for systemic and long-term transgene expression. In fact, we previously showed the efficacy of AAV1−IL-10 in rodent models of certain cardiovascular diseases^19^,^20^. The plasma IL-10 levels were markedly increased in the mice treated with AAV1−IL-10 (a vector dose of 2 × 10^11^ genome copies/mouse), whereas no IL-10 was detected in the mice treated with AAV1−GFP as a control vector (Fig. 5A). As expected, AAV1−GFP exerted no effect on Dox-induced cardiotoxicity in NLRP3^−/−^ mice; however, AAV1−IL-10 almost completely restored the cardiac dysfunction (Fig. 5B,C), occurrence of vacuolated cells (Fig. 5D), plasma levels of CPK, CK-MB, and LDH (Fig. 5E), and increased expression of *Bnp* (Fig. 5F). These results indicate that decreased IL-10 production is responsible for the enhanced susceptibility to Dox-induced cardiotoxicity in NLRP3^−/−^ mice.

## Discussion

The major findings of this study are as follows: (1) NLRP3 deficiency enhances the susceptibility to Dox-induced cardiotoxicity independently of IL-1β; (2) macrophages are the predominant inflammatory cells in the hearts; (3) IL-10 is decreased in the hearts of the Dox-treated NLRP3^−/−^ mice; (4) BMT experiments showed that bone marrow-derived cells contribute to the exacerbation of Dox-induced cardiotoxicity in the NLRP3^−/−^ mice; (5) *in vitro* experiments revealed that NLRP3 deficiency decreased IL-10 production in macrophages independently of ERK1/2, p38, and NF-κB; (6) AAV-mediated IL-10 overexpression restored the exacerbation of cardiotoxicity in the NLRP3^−/−^ mice. These findings show that NLRP3 deficiency reduces macrophage IL-10 production and enhances the susceptibility to the Dox-induced cardiotoxicity independently of IL-1β. Our study shows a novel role of NLRP3 and provides new insights into the mechanisms underlying Dox-induced cardiotoxicity.

There is increasing evidence that indicates the importance of NLRP3 inflammasomes in the pathophysiology of cardiac disease^8^; however, the role of NLRP3 inflammasomes in Dox-induced cardiotoxicity is unknown. In the present study, we hypothesized that NLRP3 inflammasomes mediate Dox-induced cardiotoxicity. Contrary to our expectations, however, NLRP3^−/−^ mice were more susceptible to the cardiotoxic effects of Dox than WT mice, and this enhanced susceptibility was not observed in IL-1β^−/−^ mice, which showed that the contribution of NLRP3 to the Dox-induced cardiotoxicity was independent of IL-1β. Inflammasome-independent functions of NLRP3 have recently received considerable attention. For instance, Shigeoka *et al.*^21^ observed that renal I/R injury was attenuated in NLRP3^−/−^ mice, but not in ASC^−/−^ and caspase-1^−/−^ mice, and concluded that NLRP3 contributed to renal I/R injury pathogenesis through an inflammasome-independent pathway. We have recently reported that NLRP3 regulates neutrophil migration and contributes to the pathophysiology of hepatic I/R injury and hyperoxia-induced lung injury independently of the inflammasome or IL-1β^16^,^17^. More recently, Bruchard *et al.*^22^ reported that NLRP3 in CD4^+^ T cells was required for differentiation of T helper type 2 cells in an inflammasome-independent manner. In the present study, we found that NLRP3 regulates IL-10 production in macrophages independently of IL-1β. To our knowledge, this study provides the first evidence that NLRP3 has cell-intrinsic roles not only in neutrophils and lymphocytes but also in macrophages.

In this study, we showed that macrophages are the predominant effector cells that mediate decreased IL-10 production in the heart; this finding is based on the following observations: (1) BMT experiments clearly showed the contribution of bone marrow-derived inflammatory cells and (2) macrophages are the predominant inflammatory cells in the hearts. Indeed, as expected, IL-10 production was decreased in macrophages from NLRP3^−/−^ mice. Intriguingly, however, Dox at the low-dose administered in this study failed to induce inflammatory cell infiltration in the hearts, which suggests the importance of cardiac-resident macrophages. Recent evidence indicates that resident macrophages in various tissues are considered to have distinct properties from monocyte-derived macrophages^23^. In this regard, it is widely accepted that monocyte-derived macrophages have an inflammatory phenotype (referred to as M1 macrophages), whereas the tissue-resident macrophages have an anti-inflammatory phenotype (referred to as M2 macrophages). In fact, cardiac-resident macrophages have an M2 phenotype characterized by production of IL-10 and transforming growth factor-β and exert cardioprotective effects under pathological conditions^24^,^25^. Another important finding to be noted here is that IL-10 overexpression attenuated the exacerbation of Dox-induced cardiotoxicity in NLRP3^−/−^ mice. Previous studies have demonstrated that IL-10 improved cardiac dysfunction and remodeling in murine models of myocardial infarction and heart failure^26^,^27^,^28^. Conversely, IL-10^−/−^ mice have shown increased infarct size and decreased survival in myocardial infarction^29^. These findings indicate the cardioprotective effect of IL-10 in cardiovascular disease. Thus, we assume that NLRP3 in the cardiac-resident macrophages regulates IL-10 production and serves to protect against Dox-induced cardiotoxicity.

Several limitations of this study should be noted. First, we clearly showed that NLRP3 deficiency decreased IL-10 production in macrophages; however, the molecular mechanism underlying NLRP3 regulation of IL-10 production remains unclear. In this regard, as described above, we showed that ERK1/2, p38, and NF-κB, which are known to be common upstream pathways for IL-10 production, is not involved in NLRP3-regulated IL-10 production. Recently, Ito *et al.*^30^ reported that Bruton’s tyrosine kinase (BTK) directly binds NLRP3 and influences the inflammasome activation in macrophages. In addition, Schmidt *et al.*^31^ showed that BTK is required for TLR-mediated IL-10 production. Therefore, we investigated the role of BTK in NLRP3-regulated IL-10 production. Consistent with the findings by Ito *et al.*, we confirmed a direct association between NLRP3 and BTK in macrophages by immunoprecipitation assay (data not shown). Unfortunately, however, pretreatment with two types of BTK inhibitors (LFM A-13 and PCI32765) significantly inhibited IL-10 production in both WT and NLRP3^−/−^ macrophages, compared with vehicle treatment (data not shown), which suggests that BTK does not contribute much to NLRP3-regulated IL-10 production. Therefore, we postulate that there may be other pathway(s) involved in IL-10 production in macrophages. Second, because we focused on the role of NLRP3 and IL-1β, the contribution of the inflammasomes still needs to be determined in mice deficient for other inflammasome components, such as ASC and caspase-1. Third, Marchetti *et al.*^32^ very recently reported that the NLRP3 inhibition ameliorated Dox-induced cardiac dysfunction and fibrosis. This is somewhat inconsistent with our findings. Although the reason for this discrepancy is unclear, differences between our study and the study by Marchetti *et al.* are the method for NLRP3 inhibition and mouse strain. We used C57BL/6 background NLRP3^−/−^ mice whereas Marchetti *et al.* used the ICR mice treated with NLRP3 inhibitor, which is an intermediate in the synthesis of glyburide. In this regard, it is known that the differences of mouse strain influences the susceptibility to drug-induced inflammatory responses^33^. Thus, further investigations are necessary to understand the precise role of NLRP3 in the pathophysiology of Dox-induced cardiotoxicity.

In conclusion, we demonstrate that NLRP3 regulates IL-10 production in macrophages and contributes to the pathophysiology of Dox-induced cardiotoxicity. Our findings identify a previously unknown function of NLRP3 that is independent of IL-1β and suggest that NLRP3 and IL-10 are potential therapeutic targets for the prevention and treatment of Dox-induced cardiotoxicity.

## Methods

### Ethics statement

The investigations conformed to the Guide for the Care and Use of Laboratory.

Animals published by the US National Institutes of Health (NIH publication, 8th Edition, 2011). The protocol was approved by the Use and Care of Experimental Animals Committee of the Jichi Medical University Guide for Laboratory Animals (permit number 15082), and were conducted in accordance with the Jichi Medical University guidelines. All efforts were made to minimize suffering animals.

### Animal protocol

NLRP3^−/−^ and IL-1β^−/−^ mice (C57BL/6J genetic background) were kindly provided by Dr. Vishava M. Dixit (Genentech, South San Francisco, CA) and Dr. Yoichiro Iwakura (Tokyo University of Science, Chiba, Japan), respectively^34^,^35^. C57BL/6J wild-type (WT) mice were purchased from Japan SLC, Inc. (Tokyo, Japan). Mice were fed, watered, and maintained on a 12-h light and dark cycle. Dox (Adryacin) was kindly gifted by Kyowa Hakko Kirin Co. Ltd., Japan. To produce Dox-induced cardiotoxicity, we intraperitoneally injected Dox (15 or 20 mg/kg) in 8–12 week-old male mice. To induce IL-10 overexpression *in vivo,* we prepared adeno-associated virus vectors encoding murine IL-10 (AAV1–IL-10) and control vectors (AAV1–green fluorescent protein [GFP])^19^. The AAV1 vectors (a vector dose of 2 × 10^11^ genome copies/mouse, 50 μL/mouse) were injected into the gastrocnemius muscle of mice 14 days prior to Dox administration.

### Echocardiography

Transthoracic echocardiography was performed at the indicated time after Dox administration using a digital ultrasound system (Vevo2100 imaging System, Visual Sonics, Toronto, Canada) with a 30-MHz probe. Briefly, after anesthetization by inhalation of 1.5% isoflurane, two-dimensional targeted M-mode echocardiograms were obtained along the short axis of the left ventricle (LV) at the level of the papillary muscles, and at least three consecutive beats were evaluated. LV end-diastolic diameter (LVEDD) and LV end-systolic diameter (LVESD), defined as the phases in which the smallest and largest area of LV, respectively, were obtained. Percentage fractional shortening (%FS) was calculated using the standard formula^36^.

### BMT

BMT mice were generated as described previously^13^. To verify the reconstitution of bone marrow after transplantation by this protocol, we used GFP mice as donors. Flow cytometric analysis showed that 8 weeks after BMT, peripheral blood cells consisted of more than 90% GFP-positive cells. Using this protocol, we produced three types of BMT^WT to WT^, BMT^WT to NLRP3−/−^, and BMT^NLRP3−/− to WT^ mice.

### Measurement for plasma enzymes and cytokines

Plasma levels of creatine phosphokinase (CPK), creatine kinase myocardial bound (CK-MB), and lactate dehydrogenase (LDH) were measured using chemical analyzer Fuji-drychem (Fuji Film, Tokyo, Japan) according to the manufacturer’s instructions. The levels of IL-1β, TNF-α, and IL-10 were assessed using a mouse enzyme-linked immunosorbent assay (ELISA) kit (R&D Systems, Minneapolis, MN).

### Real-time RT-PCR analysis

Total RNA was prepared from the heart using ISOGEN (Nippon Gene Co., Ltd., Toyama, Japan) according to the manufacturer’s instructions. Real-time RT-PCR analysis was performed using the Takara TP960 PCR Thermal Cycler Dice Detection System (Takara Bio Inc, Shiga, Japan) to detect mRNA expression. The expression levels of each target gene were normalized by subtracting the corresponding glyceraldehyde-3-phosphate dehydrogenase (GAPDH) threshold cycle (C_T_) values; normalization was carried out using the ΔΔ C_T_ comparative method. The following primers (oligonucleotide sequences are provided in parentheses in the order of sense and antisense primers) were used: *Bnp*, 5′*-*CTGAAGGTGCTGTCCCAGAT-3′ 5′-GTTCTTTTGTGAGGCCTTGG-3′; *Il1b*, 5′-TGAAGTTGACGGACCCCAAA-3′ and 5′TGATGTGCTGCTGTGAGATT-3′; *Tnfa,* 5′-CCCCAAAGGGATGAGAAGTTC-3′and 5′-GCTTGTCACTCGAATTTTGAGAA-3′; *Il10,* 5′*-*GTTGCCAAGCCTTATCGGGAA-3′ and 5′-CCAGGGAATTCAAATGCTCCT-3′, and *gapdh*, 5′-TGTGTCCGTCGTGGATCTGA-3′ and 5′-TTGCTGTTGAAGTCGCAGGAG-3′; *Bax*, 5′-TTGCTGATGGCAACTTCAAC-3′ and 5′-GATCAGCTCGGGCACTTTAG-3′; *Bcl2*, 5′-CAGAAGATCATGCCGTCCTT-3′ and 5′-CTTTCTGCTTTTTATTTCATGAGG-3′; *Tlr2*, 5′-TGGAGACGCCAGCTCTGGCTCA-3′ and 5′-CAGCTTAAAGGGCGGGTCAGAG-3′; *Tlr4,*5′-AGTGGGTCAAGGAACAGAAGCA-3′ and 5′CTTTACCAGCTCATTTCTCACC-3′.

### Histology and immunohistochemistry

The hearts were embedded in Tissue-Tek O.C.T. compound (Sakura Finetechnical Co. Ltd., Tokyo, Japan), and were cut into 4μm-thick sections and fixed with Mildform 10N (Wako Pure Chemical Industries, Ltd., Osaka). The sections were stained with hematoxylin and eosin (HE). Immunohistochemical analysis was performed to detect the pan-leukocyte marker, CD45 (BD Bioscience), and macrophage marker, CD68 (Santa Cruz). The staining sections were photographed by using a microscope (FSX-100; Olympus, Tokyo, Japan) or confocal laser scanning microscopy (FV-10i; Olympus). For quantification, the number of vacuolated cardiomyocytes, CD45^+^ cells, and CD68^+^ cells was counted in 10 randomly chosen fields at a magnification of 200× for each sample.

### Flow cytometry analysis

Cells were analyzed using flow cytometry analysis as described previously^16^. The cells were double-labeled with the following antibodies: allophycocynain (APC)-conjugated anti-CD45, fluorescein isothiocyanate (FITC)-conjugated anti-F4/80, phycoerythrin (PE)-conjugated anti-CD11b, PE-conjugated anti-Ly6G, FITC-conjugated anti-CD45R, PE-conjugated anti-CD3, FITC-conjugated anti-CD19, PE-conjugated anti-CD4, and FITC-conjugated anti-CD8. All these antibodies were obtained from eBiosceince (San Diego, CA). The cells were examined by flow cytometry (FACSCalibur; BD Biosciences) and analyzed using FlowJo software version 10 (Tree Star, Inc., San Carlos, CA). Isotype control antibodies were used as negative controls to exclude nonspecific background staining.

### Western blot analysis

Expression of IκBα, extracellular-regulated kinase1/2 (ERK1/2), p38 (p38), phospho-ERK1/2 (p-ERK1/2), phosphor-p38 (p-p38), and β-actin was analyzed by western blotting^10^. The antibodies used in this study were purchased from Cell Signaling Technology, Inc. (Danvers, MA), Santa Cruz Biotechnology (Dallas, TX), and Sigma. The expression level of β-actin served as an internal control for protein loading.

### Cell cultures and *in vitro* experiments

Murine neonatal cardiomyocytes were prepared from the ventricles of 1-day-old mice and cultured in Dulbecco’s modified Eagle’s medium (DMEM; Wako Pure Chemical Industries, Ltd.) supplemented with 10% fetal calf serum (FCS)^13^. Murine peritoneal macrophages were isolated by using the thioglycollate elicitation method and cultured in 10%FCS/DMEM. To detect IL-10 induction, cells were treated with lipopolysaccharide (LPS; Sigma) or Pam3CysSerLys4 (Pam3CSK4; Life Technologies, San Diego, CA) for the indicated period.

### Statistical analysis

Data were expressed as the mean ± standard error of the mean (SEM). An unpaired *t* test was used to compare two groups. For comparisons between multiple groups, the significance of differences in between-group means was determined by one-way analysis of variance (ANOVA) followed by the Tukey-Kramer test. All analyses were performed using the SPSS version 21 (IBM Japan Ltd., Tokyo, Japan). A *p*-value of < 0.05 was considered statistically significant.

## Additional Information

**How to cite this article**: Kobayashi, M. *et al.* NLRP3 Deficiency Reduces Macrophage Interleukin-10 Production and Enhances the Susceptibility to Doxorubicin-induced Cardiotoxicity. *Sci. Rep.* **6**, 26489; doi: 10.1038/srep26489 (2016).

## Supplementary Material

Supplementary Information
