# Supplementary material for: NLRP3 Deficiency Reduces Macrophage Interleukin-10 Production and Enhances the Susceptibility to Doxorubicin-induced Cardiotoxicity
Source: Sci Rep. 2016 May 26;6:26489. doi: 10.1038/srep26489 (PMC4880937; doi:10.1038/srep26489)
Supplement: Supplementary Information [file srep26489-s1.pdf]

NLRP3 Deficiency Reduces Macrophage Interleukin-10 Production and Enhances the Susceptibility to Doxorubicin-induced Cardiotoxicity

Motoi Kobayashi, Fumitake Usui, Tadayoshi Karasawa, Akira Kawashima, Hiroaki Kimura, Yoshiko Mizushina, Koumei Shirasuna, Hiroaki Mizukami, Tadashi Kasahara, Naoyuki Hasebe, Masafumi Takahashi

Correspondence: Masafumi Takahashi, MD, PhD, Division of Inflammation Research, Center for Molecular Medicine, Jichi Medical University
3311-1 Yakushiji, Shimotsuke, Tochigi 329-0498, Japan
E-mail: masafumi2@jichi.ac.jp

Supplementary information

Supplementary Fig. S1. Effect of high-dose Dox on cardiac dysfunction and injury

(A) Echocardiography was performed in WT and NLRP3$^{-/-}$mice 5 days after Dox (20 mg/kg) or Veh treatment. Cardiac function (%FS) was assessed (n = 6–10). (B and C) Mice were sacrificed 5 days after Dox or Veh treatment. (B) The heart sections were obtained and stained with HE. The vacuolated cardiomyocytes were quantified (n = 4 for each). (C) Plasma levels of CPK, CK-MB, and LDH were assessed (n = 5 for each). Data are expressed as the mean ± SEM. *$p < 0.05$ and **$p < 0.01$. # $p < 0.05$ vs. Veh treatment (WT).

Supplementary Fig. S2. Effect of NLRP3 deficiency on apoptosis and the expression of Bax and Bcl-2

The heart samples were obtained from WT and NLRP3$^{-/-}$ mice 5 days after Dox or Veh treatment. (A) The heart sections were stained with TUNEL staining to identify apoptotic cells. Representative photographs of TUNEL staining were shown. Arrowheads indicate TUNEL^{+} cells. Quantitative analysis of TUNEL-positive cells was performed (n = 4 for each). (B) Heart

Bax and *Bcl2* mRNA levels were assessed by real-time RT-PCR analysis (n = 4 for each). Data are expressed as the mean ± SEM.

Supplementary Fig. S3. The composition of inflammatory cells in the hearts

The composition of inflammatory cells in the hearts from WT mice was analyzed by flow cytometry. The number of neutrophils (Ly6G$^+$), macrophages (CD11b$^+$F4/80$^+$), T cells (CD4$^+$ and CD8$^+$), and B cells (CD19$^+$) in the CD45$^+$ cells was quantified (n = 5 for each). Data are expressed as the mean ± SEM.

Supplementary Fig. S4. Cardiac tissue IL-10 levels in BMT mice

BMT$^{\text{WT to WT}}$, BMT $^{\text{WT to NLRP3−/−}}$, and BMT$^{\text{NLRP3−/− to WT}}$ mice were treated with Dox or Veh 8 weeks after BMT. Cardiac tissue IL-10 levels in these BMT mice were assessed (n =4). **$p < 0.01$. # $p < 0.05$ vs. Veh treatment (BMT$^{\text{WT to WT}}$).

Supplementary Fig. S5. Effect of NLRP3 deficiency on IκBα degradation and phosphorylation of ERK1/2 and p38

Primary macrophages derived from WT and NLRP3$^{-/-}$ mice were stimulated with LPS (100 ng/mL) for the indicated periods. (A) Expression of IκBα and phosphorylation of ERK1/2 (p-ERK1/2) and p38 (p-p38) was analyzed by western blotting. Total protein of ERK1/2, p38, and β-actin was shown as a reference of equal loading. (B) Nuclear translocation was detected by immunofluorescence staining. Nucleic acid was stained with DAPI.

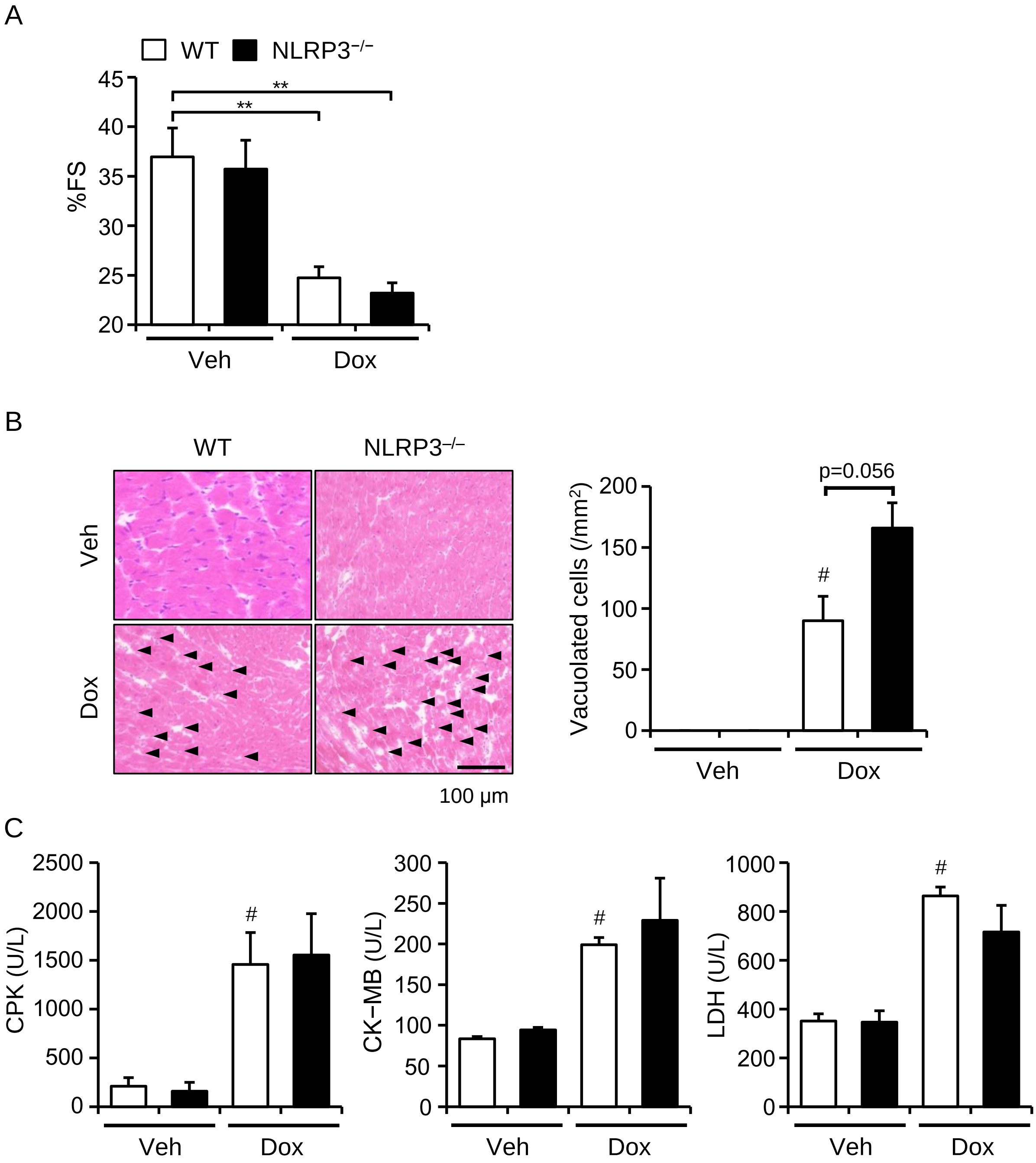
A
WT
NLRP3−/−
%FS
45
40
35
30
25
20
**
**
Veh
Dox
B
WT
NLRP3−/−
Veh
Dox
100 μm
Vacuolated cells (/mm2)
200
150
100
50
0
p=0.056
#
Veh
Dox
C
CPK (U/L)
2500
2000
1500
1000
500
0
#
Veh
Dox
CK−MB (U/L)
300
250
200
150
100
50
0
#
Veh
Dox
LDH (U/L)
1000
800
600
400
200
0
#
Veh
Dox

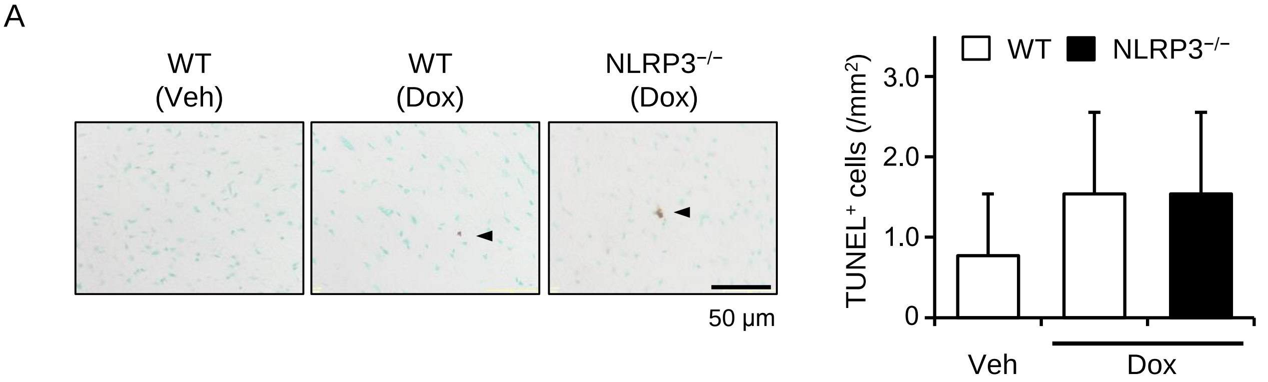
A
WT
(Veh)
WT
(Dox)
NLRP3–/–
(Dox)
50 μm
WT
NLRP3–/–
TUNEL+ cells (/mm2)
3.0
2.0
1.0
0
Veh
Dox

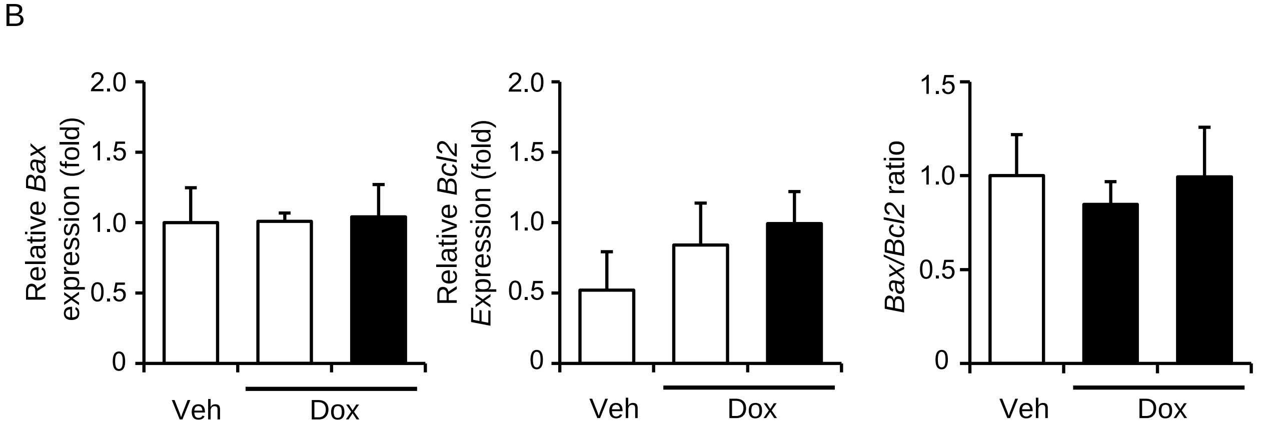
B
Relative Bax expression (fold)
2.0
1.5
1.0
0.5
0
Veh
Dox
Relative Bcl2 Expression (fold)
2.0
1.5
1.0
0.5
0
Veh
Dox
Bax/Bcl2 ratio
1.5
1.0
0.5
0
Veh
Dox

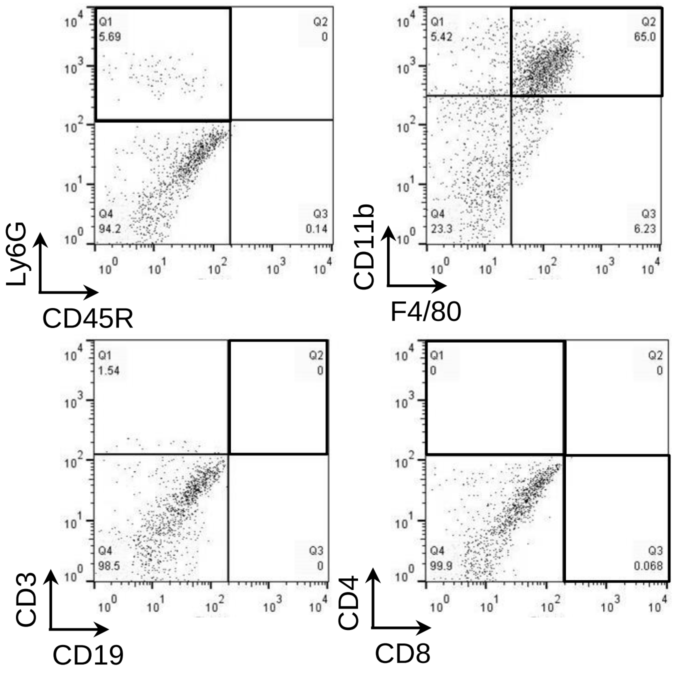
Q1
5.69
Q2
0
Q4
94.2
Q3
0.14
Ly6G
CD45R
Q1
5.42
Q2
65.0
Q4
23.3
Q3
6.23
CD11b
F4/80
Q1
1.54
Q2
0
Q4
98.5
Q3
0
CD3
CD19
Q1
0
Q2
0
Q4
99.9
Q3
0.068
CD4
CD8

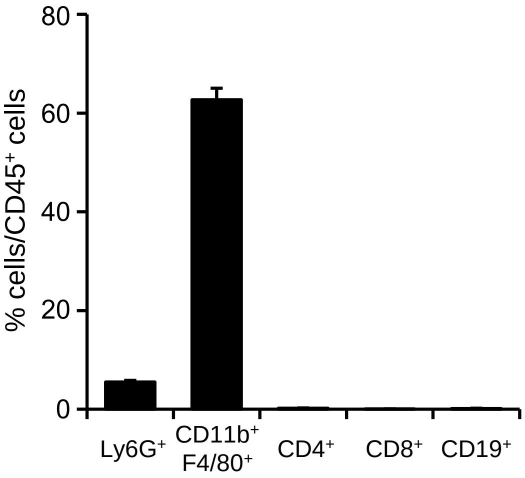
% cells/CD45⁺ cells
80
60
40
20
0
Ly6G⁺
CD11b⁺ F4/80⁺
CD4⁺
CD8⁺
CD19⁺

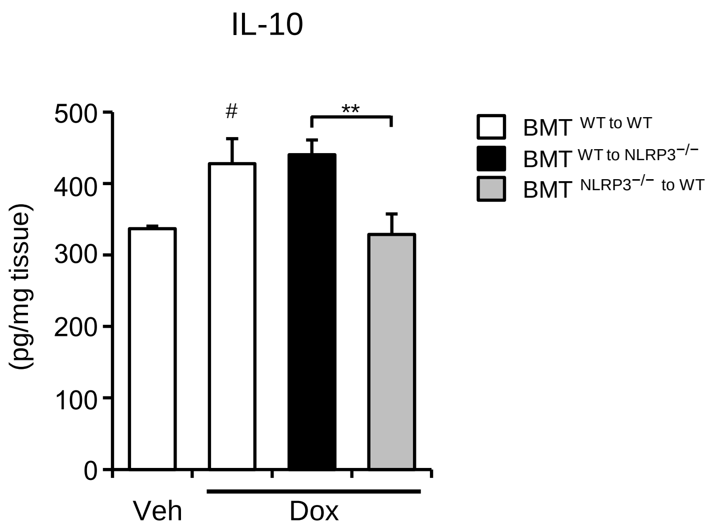
IL-10
500
400
300
200
100
0
(pg/mg tissue)
#
**
Veh
Dox
BMT WT to WT
BMT WT to NLRP3−/−
BMT NLRP3−/− to WT

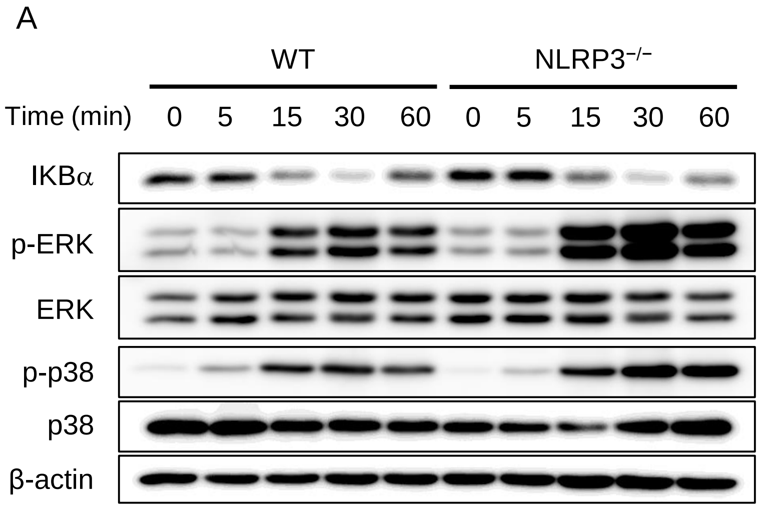
A
WT
NLRP3−/−
Time (min)
0
5
15
30
60
0
5
15
30
60
IKBα
p-ERK
ERK
p-p38
p38
β-actin

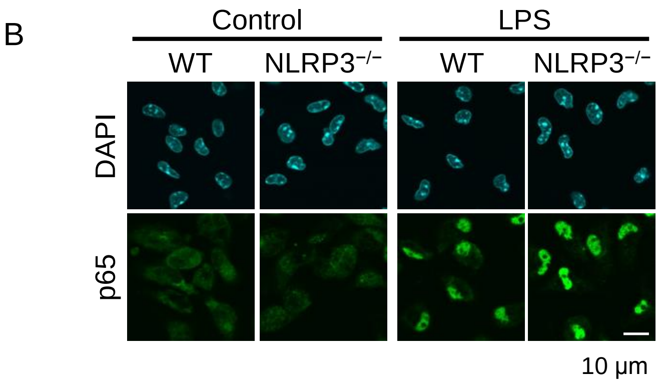
B
Control
LPS
WT
NLRP3−/−
WT
NLRP3−/−
DAPI
p65
10 μm
